# mikropml: User-Friendly R Package for Supervised Machine Learning Pipelines

**DOI:** 10.21105/joss.03073

**Published:** 2021-05-14

**Authors:** Begüm D. Topçuoğlu, Zena Lapp, Kelly L. Sovacool, Evan Snitkin, Jenna Wiens, Patrick D. Schloss

**Affiliations:** 1 Department of Computational Medicine & Bioinformatics, University of Michigan; 2 Department of Electrical Engineering & Computer Science, University of Michigan; 3 Department of Microbiology & Immunology, University of Michigan; 4 Exploratory Science Center, Merck & Co., Inc., Cambridge, Massachusetts, USA.; 5 Department of Internal Medicine/Division of Infectious Diseases, University of Michigan

## Abstract

Machine learning (ML) for classification and prediction based on a set of features is used to make decisions in healthcare, economics, criminal justice and more. However, implementing an ML pipeline including preprocessing, model selection, and evaluation can be time-consuming, confusing, and difficult. Here, we present mikropml (prononced “meek-ROPE em el”), an easy-to-use R package that implements ML pipelines using regression, support vector machines, decision trees, random forest, or gradient-boosted trees. The package is available on GitHub, CRAN, and conda.

## Graphical Abstract

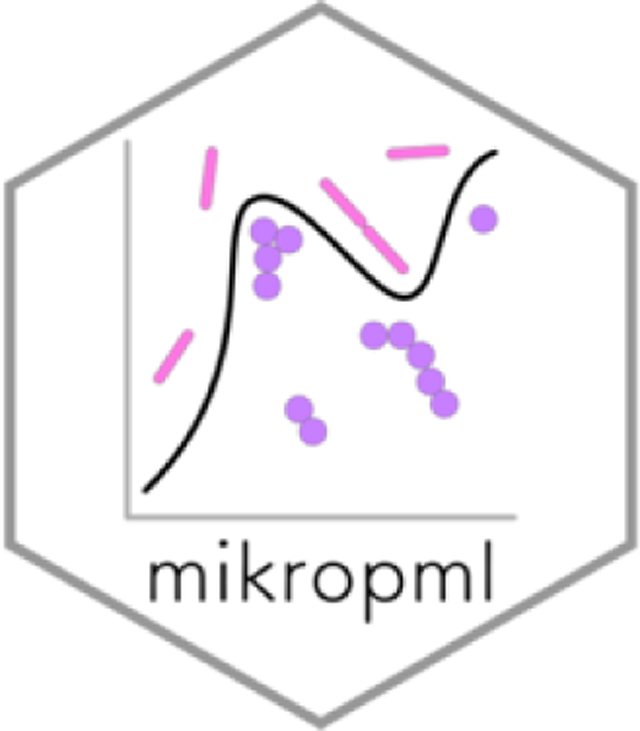

## Statement of need

Most applications of machine learning (ML) require reproducible steps for data pre-processing, cross-validation, testing, model evaluation, and often interpretation of why the model makes particular predictions. Performing these steps is important, as failure to implement them can result in incorrect and misleading results (Teschendorff, 2019; Wiens et al., 2019).

Supervised ML is widely used to recognize patterns in large datasets and to make predictions about outcomes of interest. Several packages including caret (Kuhn, 2008) and tidymod els (Kuhn et al., 2020) in R, scikitlearn (Pedregosa et al., 2011) in Python, and the H2O autoML platform (H2O.ai, 2020) allow scientists to train ML models with a variety of algorithms. While these packages provide the tools necessary for each ML step, they do not implement a complete ML pipeline according to good practices in the literature. This makes it difficult for practitioners new to ML to easily begin to perform ML analyses.

To enable a broader range of researchers to apply ML to their problem domains, we created mikropml, an easy-to-use R package (R Core Team, 2020) that implements the ML pipeline created by Topçuoğlu *et al.* (Topçuoğlu et al., 2020) in a single function that returns a trained model, model performance metrics and feature importance. mikropml leverages the caret package to support several ML algorithms: linear regression, logistic regression, support vector machines with a radial basis kernel, decision trees, random forest, and gradient boosted trees. It incorporates good practices in ML training, testing, and model evaluation (Teschendorff, 2019; Topçuoğlu et al., 2020). Furthermore, it provides data preprocessing steps based on the FIDDLE (FlexIble Data-Driven pipeLinE) framework outlined in Tang *et al.* (Tang et al., 2020) and post-training permutation importance steps to estimate the importance of each feature in the models trained (Breiman, 2001; Fisher et al., 2018).

mikropml can be used as a starting point in the application of ML to datasets from many different fields. It has already been applied to microbiome data to categorize patients with colorectal cancer (Topçuoğlu et al., 2020), to identify differences in genomic and clinical features associated with bacterial infections (Lapp et al., 2020), and to predict gender-based biases in academic publishing (Hagan et al., 2020).

## mikropml package

**Figure 1:**
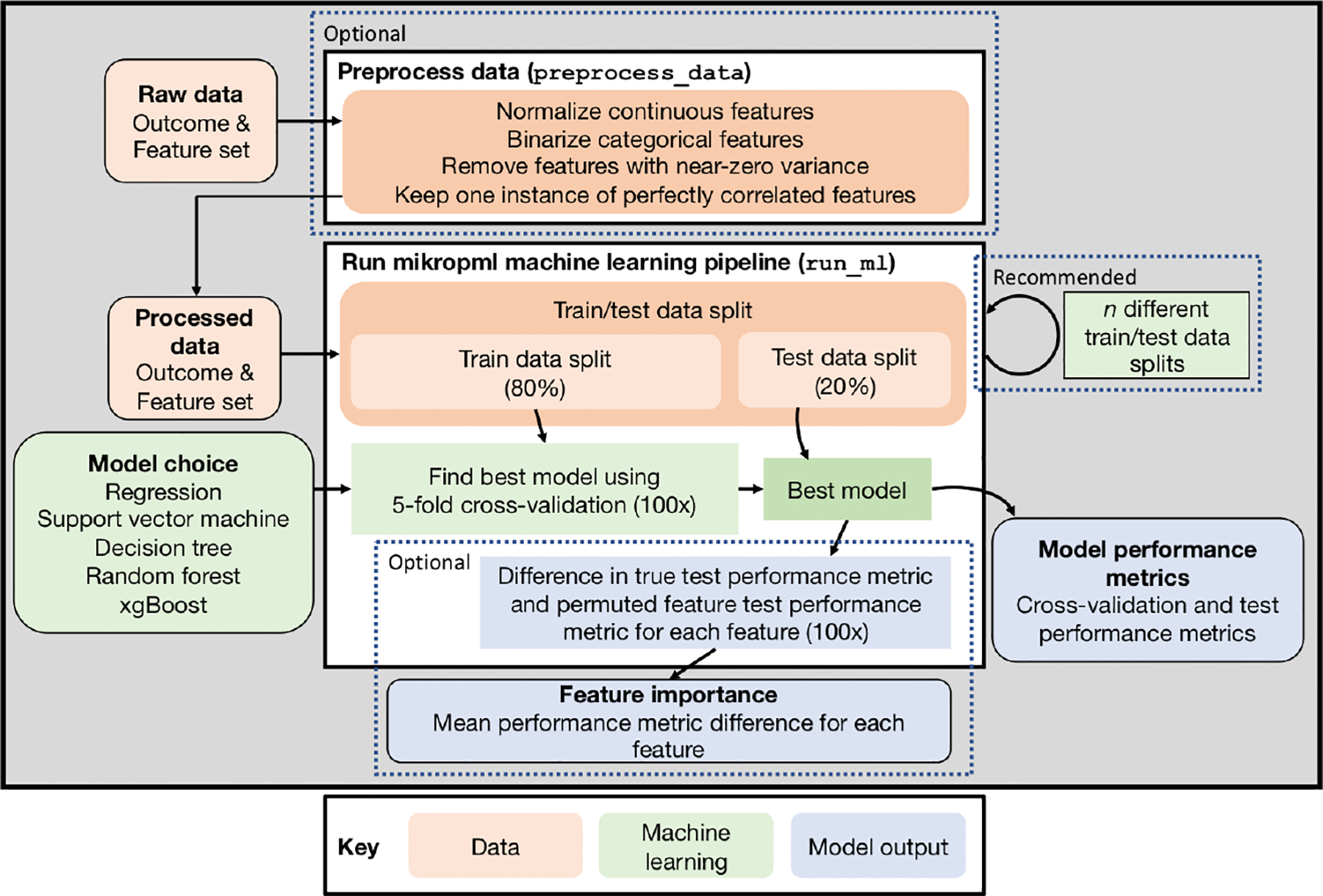
mikropml pipeline

The mikropml package includes functionality to preprocess the data, train ML models, evaluate model performance, and quantify feature importance (Figure 1). We also provide vignettes and an example Snakemake workflow (Köster & Rahmann, 2012) to showcase how to run an ideal ML pipeline with multiple different train/test data splits. The results can be visualized using helper functions that use ggplot2 (Wickham, 2016).

While mikropml allows users to get started quickly and facilitates reproducibility, it is not a replacement for understanding the ML workflow which is still necessary when interpreting results (Pollard et al., 2019). To facilitate understanding and enable one to tailor the code to their application, we have heavily commented the code and have provided supporting documentation which can be read online.

## Preprocessing data

We provide the function preprocess_data() to preprocess features using several different functions from the caret package. preprocess_data() takes continuous and categorical data, re-factors categorical data into binary features, and provides options to normalize continuous data, remove features with near-zero variance, and keep only one instance of perfectly correlated features. We set the default options based on those implemented in FIDDLE (Tang et al., 2020). More details on how to use preprocess_data() can be found in the accompanying vignette.

## Running ML

The main function in mikropml, run_ml(), minimally takes in the model choice and a data frame with an outcome column and feature columns. For model choice, mikropml currently supports logistic and linear regression (glmnet: Friedman et al., 2010), support vector machines with a radial basis kernel (kernlab: Karatzoglou et al., 2004), decision trees (rpart: Therneau et al., 2019), random forest (randomForest: Liaw & Wiener, 2002), and gradient-boosted trees (xgboost: Chen et al., 2020). run_ml() randomly splits the data into train and test sets while maintaining the distribution of the outcomes found in the full dataset. It also provides the option to split the data into train and test sets based on categorical variables (e.g. batch, geographic location, etc.). mikropml uses the caret package (Kuhn, 2008) to train and evaluate the models, and optionally quantifies feature importance. The output includes the best model built based on tuning hyperparameters in an internal and repeated cross-validation step, model evaluation metrics, and optional feature importances. Feature importances are calculated using a permutation test, which breaks the relationship between the feature and the true outcome in the test data, and measures the change in model performance. This provides an intuitive metric of how individual features influence model performance and is comparable across model types, which is particularly useful for model interpretation (Topçuoğlu et al., 2020). Our introductory vignette contains a comprehensive tutorial on how to use run_ml().

## Ideal workflow for running mikropml with many different train/test splits

To investigate the variation in model performance depending on the train and test set used (Lapp et al., 2020; Topçuoğlu et al., 2020), we provide examples of how to run_ml() many times with different train/test splits and how to get summary information about model performance on a local computer or on a high-performance computing cluster using a Snakemake workflow.

## Tuning & visualization

One particularly important aspect of ML is hyperparameter tuning. We provide a reasonable range of default hyperparameters for each model type. However practitioners should explore whether that range is appropriate for their data, or if they should customize the hyperparameter range. Therefore, we provide a function plot_hp_performance() to plot the cross-validation performance metric of a single model or models built using different train/test splits. This helps evaluate if the hyperparameter range is being searched exhaustively and allows the user to pick the ideal set. We also provide summary plots of test performance metrics for the many train/test splits with different models using plot_model_performance(). Examples are described in the accompanying vignette on hyperparameter tuning.

## Dependencies

mikropml is written in R (R Core Team, 2020) and depends on several packages: dplyr (Wickham et al., 2020), rlang (Henry et al., 2020) and caret (Kuhn, 2008). The ML algorithms supported by mikropml require: glmnet (Friedman et al., 2010), e1071 (Meyer et al., 2020), and MLmetrics (Yan, 2016) for logistic regression, rpart2 (Therneau et al., 2019) for decision trees, randomForest (Liaw & Wiener, 2002) for random forest, xgboost (Chen et al., 2020) for xgboost, and kernlab (Karatzoglou et al., 2004) for support vector machines. We also allow for parallelization of cross-validation and other steps using the foreach, doFuture, future.apply, and future packages (Bengtsson & Team, 2020). Finally, we use ggplot2 for plotting (Wickham, 2016).
